# Utility of a single adjusting compartment: a novel methodology for whole body physiologically-based pharmacokinetic modelling

**DOI:** 10.1186/1742-4682-5-19

**Published:** 2008-08-08

**Authors:** Hirotaka Ando, Shigeru Izawa, Wataru Hori, Ippei Nakagawa

**Affiliations:** 1 Discovery Research Laboratories, Kyorin Pharmaceutical Co., Ltd., Tochigi, Japan; 2 PM Office Research Headquarters, Kyorin Pharmaceutical Co., Ltd., Tokyo, Japan

## Abstract

**Background:**

There are various methods for predicting human pharmacokinetics. Among these, a whole body physiologically-based pharmacokinetic (WBPBPK) model is useful because it gives a mechanistic description. However, WBPBPK models cannot predict human pharmacokinetics with enough precision. This study was conducted to elucidate the primary reason for poor predictions by WBPBPK models, and to enable better predictions to be made without reliance on complex concepts.

**Methods:**

The primary reasons for poor predictions of human pharmacokinetics were investigated using a generic WBPBPK model that incorporated a single adjusting compartment (SAC), a virtual organ compartment with physiological parameters that can be adjusted arbitrarily. The blood flow rate, organ volume, and the steady state tissue-plasma partition coefficient of a SAC were calculated to fit simulated to observed pharmacokinetics in the rat. The adjusted SAC parameters were fixed and scaled up to the human using a newly developed equation. Using the scaled-up SAC parameters, human pharmacokinetics were simulated and each pharmacokinetic parameter was calculated. These simulated parameters were compared to the observed data. Simulations were performed to confirm the relationship between the precision of prediction and the number of tissue compartments, including a SAC.

**Results:**

Increasing the number of tissue compartments led to an improvement of the average-fold error (AFE) of total body clearances (CL_tot_) and half-lives (T_1/2_) calculated from the simulated human blood concentrations of 14 drugs. The presence of a SAC also improved the AFE values of a ten-organ model from 6.74 to 1.56 in CL_tot_, and from 4.74 to 1.48 in T_1/2_. Moreover, the within-2-fold errors were improved in all models; incorporating a SAC gave results from 0 to 79% in CL_tot_, and from 14 to 93% in T_1/2 _of the ten-organ model.

**Conclusion:**

By using a SAC in this study, we were able to show that poor prediction resulted mainly from such physiological factors as organ blood flow rate and organ volume, which were not satisfactorily accounted for in previous WBPBPK models. The SAC also improved precision in the prediction of human pharmacokinetics. This finding showed that the methodology of our study may be useful for functionally reinforcing a WBPBPK model.

## Background

Various methods have been developed for predicting human pharmacokinetics, including Dedrick's approach, non-compartment analysis, and an in vitro-in vivo extrapolation (IVIVE) approach used for drug discovery. Dedrick's approach is an animal scaling-up method, which is used to extrapolate human pharmacokinetic parameters from at least 2 animal species [1,2]. In contrast, the IVIVE approach, which is also used to extrapolate clinical pharmacokinetic parameters, uses in vitro materials such as hepatocytes and microsomes to scale up to an actual target pharmacokinetic parameter such as organ clearance [3,4]. Among these options, two different models have been used for many years. The compartment model, which has a long history, is still the preferred choice because it is easy to apply. However, this approach consumes considerable resources when an animal scale-up approach is used, as many animal experiments are required for proper analysis; also, the range of application is limited [5]. In contrast, whole body physiologically-based pharmacokinetic (WBPBPK) models for simulating human pharmacokinetics [6] enable the time-course of the tissue concentrations of various drugs to be simulated using data from only one species. A WBPBPK model can also be used for pharmacokinetic/pharmacodynamic (PK/PD) analysis at a target site. However, such models have not been commonly used because they are complex. Thus, it would be advantageous to develop a WBPBPK model based on a simple concept that is easy to implement.

WBPBPK models have been much investigated. They exhibit comparatively satisfactory precision in predicting human pharmacokinetics [7,8]. They are generic, consisting of already well-known methods applicable to rational PK/PD simulation. However, they do not include solutions for correction when the data used as input parameters show considerable divergence (e.g. as a result of factors associated with in vitro and in vivo studies). Therefore, improvement in the precision of predictions cannot be expected from previous models. Recently, several WBPBPK models have also been analyzed using a single simplified method [9]. Unfortunately, the more simplified versions do not account for the complexity of biological systems, as mixed models consist of individual organs as well as multiple organs considered together. Thus, it has remained difficult to apply PK/PD analysis at the level of a target organ, although this method can be useful since it is relatively simple.

It remains desirable to develop a generic, simple, and more precise WBPBPK model that is useful at the preclinical stage. Although generic WBPBPK models satisfy the conditions mentioned above (i.e. they can apply to PK/PD analysis), the ones currently in use are difficult to apply to the analysis of various compounds owing to poor predictive precision and the lack of solutions for correction. However, if these faults could be rectified, the generic WBPBPK model would be a more useful method. To improve the precision of prediction, it is important to use the available experimental data more efficiently. For example, preclinical in vivo experiments on rats are essential for Investigating New Drug (IND) applications. Such data are useful for predicting human pharmacokinetics using the generic WBPBPK model, even when the findings are derived from in silico or in vivo experiments [10]. They should ideally be used prior to the initiation of clinical trials by the pharmaceutical industry. However, it is possible that the aforementioned data are insufficient for satisfactory prediction, because a more convenient supplementary method for improving the precision of human pharmacokinetics prediction with only slight modifications is not currently available.

The aim of the present study was to construct a WBPBPK model that will enable human pharmacokinetics to be predicted with high precision using only in vivo data from rat studies and in vitro data from liver microsomes or hepatocytes, and will be supplemented by straightforward mathematical methods devoid of highly complex concepts. We also used the method developed here indirectly to investigate the potential reasons why the predictions achieved to date with precursors of the method have been poor. To these ends, we used the following procedures. 1. We speculated about the possible causes of poor precision of prediction and changed part of a generic WBPBPK model accordingly. 2. We developed a novel method and deployed it to identify and ameliorate the causes of poor prediction. The utility of the new method was demonstrated by comparing the precision with which it predicted pharmacokinetic parameters to evaluate its validity. 3. We elucidated the causes of poor precision of prediction using the developed method. Because this method involves only physiology-related parameters, it can show whether any of these parameters contribute to the lack of precision in prediction. This is the first investigation aimed at improving the precision of prediction by WBPBPK models by attempting to elucidate the reasons for the lack of such precision.

## Materials and methods

### Experimentation and data collection

Fourteen drugs with various physicochemical properties were selected for this study. Tolbutamide [11-13] and diclofenac [12,14-19] were used as acidic drugs. Midazolam [12,20-22] and diazepam [12,23,24] were used as neutral drugs. Phenytoin [11,12,14,25,26], imipramine [12,27-30] and lidocaine [12,31-35] were used as basic drugs. Gatifloxacin [36], grepafloxacin [37-39], gemifloxacin [40,41], pazufloxacin [38,42-45], enoxacin [38,46-48], fleroxacin [36,38,49] and lomefloxacin [50,51] were used as zwitterionic drugs. Data collected from the published literature about these drugs are shown in Table 1. Kp values (steady state tissue-plasma partition coefficients) were also obtained from the literature and are described in the reference column of Table 1. Physicochemical parameters such as molecular weight (M.W.), calculated logP (clogP), topological polar surface area (tPSA) and calculated molecular reflectability (cMR) were determined using ChemOffice Ultra 9.0 (Cambridge Software, USA).

**Table 1:** Pharmacokinetic parameters of various compounds used as inputs for each WBPBPK model simulation

Compound	Rat							Human				References
			
	CL_tot_ (mL/h/kg)	CL_h_ (mL/h/kg)	CL_r_ (mL/h/kg)	CL_s_ (mL/h/kg)	T_1/2_ (h)	R_B_^a^	f_B_	CL_tot_ (mL/h/kg)	T_1/2_ (h)	R_B_^*a*^	f_B_	
Tolbutamide	109	109	0	0	1.8	0.75	0.36	24.0	7.0	0.75	0.12	11–13
Diclofenac	1809	1176	633	0	0.14	0.55	0.009	447	1.2	0.55	0.009	12, 14–19
Midazolam	3024	1542	269	1213	0.53	1	0.066	473	2.8	0.80	0.043	12, 20–22
Diazepam	2492	2343	149	0	1.1	1.04	0.13	40.4	32.8	1.04	0.03	12, 23, 24
Phenytoin	1806	1246	181	379	0.37	0.99	0.23	187	13.2	0.61	0.20	11, 12, 14, 25, 26
Imipramine	2544	1649	895	0	3.5	1.67	0.01	424	16.5	1.67	0.14	12, 27–30
Lidocaine	4252	1276	2764	213	0.57	1.27	0.30	938	2.1	0.80	0.81	12, 31–35
Gatifloxacin	1101	341	574	186	1.8	1.07	0.68	252	6.5	1.07	0.75	36
Grepafloxacin	1079	917	151	11	3.4	1.34	0.44	245	11.6	1.1	0.45	37–39
Gemifloxacin	1300	163	599	432	1.6	1	0.57	500	7.0	1.2	0.30	40, 41
Pazufloxacin	970	90	721	159	0.88	1	0.74	384	1.8	1	0.77	38, 42–45
Enoxacin	1794	57	940	797	1.8	0.91	0.71	527	6.0	0.91	0.57	38, 46–48
Fleroxacin	285	57	195	34	2.6	1.29	0.40	120	9.5	1	0.77	36, 38, 49
Lomefloxacin	1243	973	270	0	4.0	1	0.69	252	7.1	1	0.79	50, 51

^*a*^R_B _(blood-plasma concentration ratio) assumed to be 1 when there were no data in the literature.

All the observed human data in this study were obtained from the literature and were used as published or with the proper corrections. The total plasma clearance was corrected to the total blood clearance using the blood-plasma concentration ratio for calculations.

### Model development

#### Generic WBPBPK model

**Figure 1:**
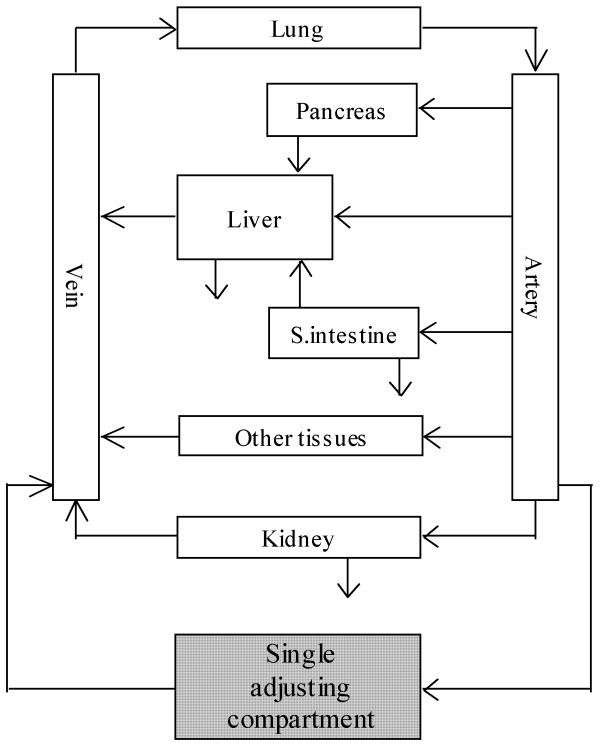
**Concept of the SAC-WBPBPK model**. The compartment "other organs" contained brain, muscle, adipose tissue and bone. Pancreas and bone were not incorporated in the 8-organ model, and adipose tissue and muscle were omitted from the 6-organ model.

The simple WBPBPK model without membrane permeation was used (equations 1–7). This model incorporated veins (v), arteries (a), lung, pancreas (panc), heart, liver (h), kidney (r), small intestine (gi), brain, adipose tissue, muscle and bone, as well as a single adjusting compartment (Figure 1). The well-stirred model was used for modelling each organ and tissue type. The rat Kp values were used without correction. Organ clearance was used to describe system clearance. It was assumed that the excreting organs were the liver, kidney and small intestine. Physiological input parameters (e.g. the blood flow rate in each organ or tissue [Q_i_] and the volume of the organ or tissue [V_i_]) were obtained from the literature [52].

A system of three ordinary linear differential equations was proposed for liver, kidney and small intestine, which are organs with elimination processes such as metabolism and excretion of bile and urine. The following equations were used [7]:

(1) $$\frac{dC_{h}}{dt}=\frac{C_{a}\left(Q_{h}-Q_{gi}-Q_{panc}\right)}{V_{h}}+\frac{Q_{gi}\cdot C_{gi}}{V_{h}\cdot Kp_{gi}}+\frac{Q_{panc}\cdot C_{panc}}{V_{h}\cdot Kp_{panc}}-\frac{Q_{h}\cdot C_{h}}{V_{h}\cdot Kp_{h}}-\frac{C_{a}\left(Q_{h}\cdot f_{B}\cdot CL_{\mathrm{int},h}\right)}{V_{h}\left(Q_{h}+f_{B}\cdot CL_{\mathrm{int},h}\right)}$$

(2) $$\frac{dC_{r}}{dt}=\frac{Q_{r}\left(C_{a}-C_{r}\right)}{V_{r}\cdot Kp_{r}}-\frac{CL_{r}\cdot C_{a}}{V_{r}}$$

(3) $$\frac{dC_{gi}}{dt}=\frac{Q_{gi}\left(C_{a}-C_{gi}\right)}{V_{gi}\cdot Kp_{gi}}-\frac{CL_{gi}\cdot C_{a}}{V_{gi}}$$

where C is the concentration, Q is the blood flow rate, V is the volume of tissue or organ, and Kp is the steady-state tissue-plasma partition coefficient.

Another system of linear ordinary differential equations was proposed for the lung and other organs, including a single adjusting compartment, with no elimination process. The following equations were used:

(4) $$\frac{dC_{lung}}{dt}=\frac{Q_{tot}\left(C_{v}-C_{lung}\right)}{V_{lung}\cdot Kp_{lung}}$$

(5) $$\frac{dC_{i}}{dt}=\frac{Q_{i}\left(C_{a}-C_{i}\right)}{V_{i}\cdot Kp_{i}}$$

where i represents the other organ.

Two linear ordinary differential equations were proposed for veins and arteries, and the following equations were used:

(6) $$\frac{dC_{v}}{dt}=\sum \left(\frac{Q_{i}\cdot C_{i}}{V_{v}\cdot Kp_{i}}\right)-\frac{Q_{tot}\cdot C_{v}}{V_{v}}$$

(7) $$\frac{dC_{a}}{dt}=\left(\frac{C_{lung}}{Kp_{lung}}-C_{a}\right)\frac{Q_{tot}}{V_{a}}$$

Pancreas and bone were not incorporated in the 8-organ model, and the adipose tissue and muscle were omitted from the 6-organ model.

The system of linear ordinary differential equations describing the WBPBPK model was solved numerically using the Runge-Kutta-Gill method [53].

A correction for intrinsic clearance in the liver was performed for acidic, neutral and basic compounds, using the in vitro intrinsic liver clearance of both rats and humans [12]. This correction was necessary because of the large species differences in metabolism. The following equation was used for scaling up from the rat to the human model:

(8) $$CL_{\mathrm{int},human,invivo}=CL_{\mathrm{int},rat,invivo}\left(\frac{CL_{\mathrm{int},human,invitro}}{CL_{\mathrm{int},rat,invitro}}\right)\cdot sf$$

In this equation, sf represents a scaling factor, and the human:rat hepatic blood flow rate ratio was taken as 0.325.

Renal and secretion clearance corrections for the blood flow were performed for scaling up from a rat model to a human model because it has been reported that blood flow rate is useful for correcting some pharmacokinetic parameters [54-56]:

(9) $$CL_{org,human}=CL_{org,rat}\left(\frac{Q_{j,human}}{Q_{j,rat}}\right)$$

where CL_org _represents clearance in the kidney or small intestine, and Q_j _represents the blood flow rate in these organs.

### Single adjusting compartment

A single adjusting compartment (SAC) was incorporated into the present model as a potential function that can offset the lack of predictive precision. The SAC was incorporated as a newly-developed virtual organ possessing the same functions as other organs in place of the "rest of the body" (carcass) previously used in WBPBPK modelling. However, the physiological parameters of the SAC were set up so that they could be adjusted arbitrarily. It was assumed that the lack of precision in simulating human pharmacokinetics has typically been caused by certain physiological factors. Thus, to describe the SAC, its blood flow rate (Q_SAC_), organ/tissue volume (V_SAC_) and steady-state tissue-plasma partition coefficient (Kp_SAC_) were selected as input parameters. The SAC was also described using the well-stirred model (equation 5). Simulated rat pharmacokinetics were fitted to the observed pharmacokinetics using Q_SAC_, V_S _and a Kp_SAC_, all of which could be changed arbitrarily. These SAC values used for fitting were fixed as data derived from rat studies.

When the Q_SAC _of a rat was transformed to a human value, the following equation was used:

(10) $$Q_{SAC,human}=Q_{SAC,rat}\left[1-\sum \left(\frac{P}{Q_{ri}}\right)\right]\cdot \frac{Q_{tot,human}}{Q_{tot,rat}}$$

where Q_ri _is the blood flow rate in the isolated organ. P is a factor that depends on the individual model; P = 15 was used for this study. This value was fixed after optimising the 6- and 8-organ model simulations for correcting the Q_SAC, rat _where the values were lager than the human Q_tot_. This value is intrinsically different for each compound, but was assumed to be constant in order to give the model generality.

The following equation was used to transform rat to human V_SAC_:

(11) $$V_{SAC,human}=V_{SAC,rat}\frac{\sum V_{i,human}}{\sum V_{i,rat}}$$

Veins and arteries were not incorporated into the total volume for each organ or tissue in a SAC. In addition, Kp_SAC_, which was used as a parameter to describe the tissue distribution of a SAC, was assumed to be the same as the value obtained from the rat. This method was used as an alternative compartment in place of the "rest of the body". The ability to be arbitrary is its main advantage. In contrast, the "rest of the body" has only a fixed parameter, which could be a major cause of poor prediction.

### Calculation of pharmacokinetic parameters

In general, the half-life (T_1/2_) and the total clearance (CL_tot_) are used to compare the precision of prediction of human pharmacokinetics among models [7-9]. Therefore, we used these parameters for this purpose. The T_1/2 _was calculated using equation 12, and k_el _(the terminal phase rate constant) was obtained by linear regression analysis of the log-transformed concentration-time data. The total area under the blood concentration-time curve (AUC_inf_) was obtained according to the following procedure. Blood AUC_0-t _values (where t is the time of the last blood concentration collected) were estimated using Simpson's rule [57], a more reasonable method than the trapezoidal method for calculating the AUC precisely. AUC_t-inf _was estimated by dividing the final blood concentration measured by the terminal-phase rate constant. AUC_inf _is the sum of AUC_0-t _and AUC_t-inf_. CL_tot _was calculated according to equation 13.

(12) $$T_{1/2}=\frac{\ln2}{k_{el}}$$

(13) $$CL_{tot}=\frac{Dose}{AUC_{\inf}}$$

### Statistical analysis

The accuracy and precision of the calculated values were confirmed by considering the ratio of the observed to the predicted values. Average values were used to confirm accuracy, and the average-fold error (AFE) [24] and the within-2-fold error were used to confirm precision. The AFE was calculated using the following equation:

(14) $$AFE=10^{\frac{1}{N}\sum \left|\log\left(\frac{observed}{simulated}\right)\right|}$$

where N represents the number of data inputs used for the calculation.

In order to clarify the major cause of poor predictions by WBPBPK models, we confirmed the correlations between certain SAC input parameters and various physicochemical parameters, which were calculated on the basis of the structures of the selected compounds.

## Results

**Figure 2:**
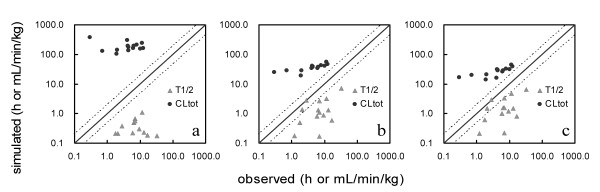
**Correlation between the observed and simulated pharmacokinetic parameters predicted without a SAC**. (a) Six-organ model without a SAC, (b) 8-organ model without a SAC, (c) 10-organ model without a SAC. The solid line represents unity, whereas the dashed lines represent the 2-fold prediction error.

**Figure 3:**
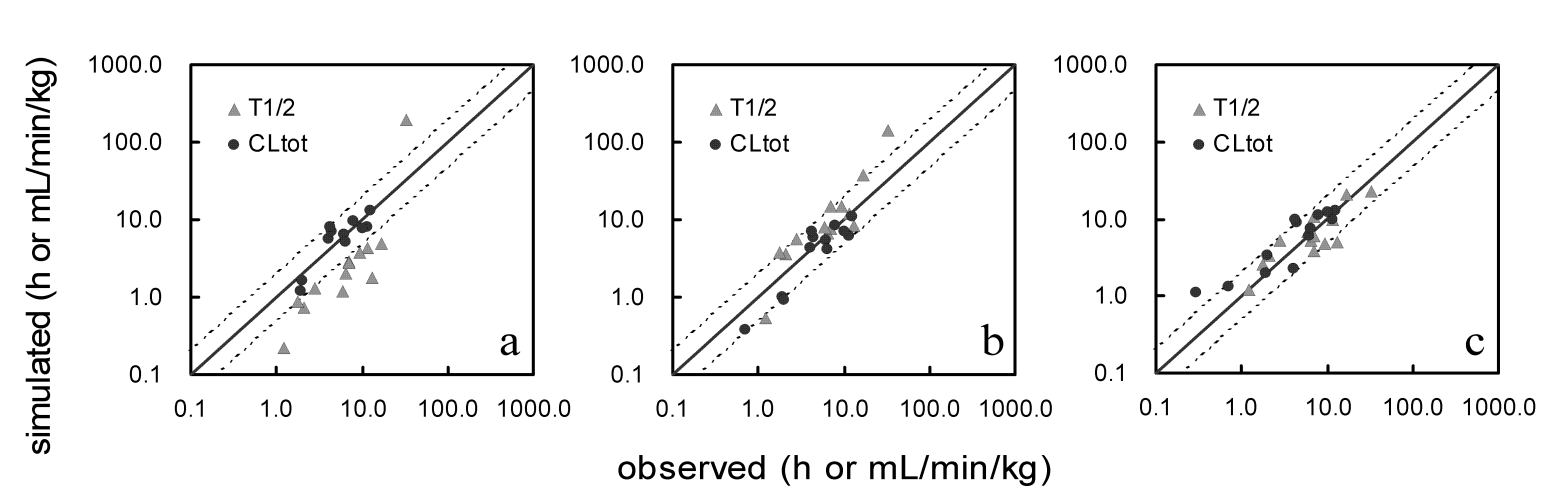
**Correlation between the observed and simulated pharmacokinetic parameters predicted with a SAC**. (a) Six-organ model with a SAC, (b) 8-organ model with a SAC, (c) 10-organ model with a SAC. The solid line represents unity, whereas the dashed lines represent the 2-fold prediction error.

A generic WBPBPK model and the single adjusting compartment (SAC)-WBPBPK model were constructed with parameters that depended on each compound. The precision of predictions was confirmed for each model. The influence of the following two factors on the precision of simulation of human pharmacokinetics was investigated: the number of organs incorporated and the presence or absence of a SAC. The human blood concentration of each compound was simulated using the constructed model. The half-life (T_1/2_) and total clearance (CL_tot_) values were calculated from the simulated human blood concentration. Figure 2a–c shows the relationship of the observed and predicted CL_tot _and T_1/2 _values when a SAC was not incorporated and the number of organs changed. The predicted values differed widely from the observed values. No satisfactory improvement in divergence was observed in spite of the addition of organs. Figure 3a–c shows the relationship observed when a SAC was incorporated and the number of organs altered. The predicted values resembled the observed values more closely in the model incorporating a SAC than in the models lacking a SAC. The precision of the simulated values in each model was confirmed by comparing the average fold error (AFE) and the within-2-fold error. These results (Table 2) showed that the precision of predictions of human T_1/2 _values decreased when some organs were removed from the model, regardless of the incorporation of a SAC. In the case of CL_tot_, the SAC-incorporated model yielded highly precise predictions in each of the three organ-number models, even the 6-organ model; the within 2-fold error was 92%. The AFE and within-2-fold error values were compared to those obtained from previous generic WBPBPK models and with those obtained by the conventional method for predicting human pharmacokinetics (Table 3). The predictions obtained with the SAC-WBPBPK model were more precise than those yielded by the other models.

**Table 2:** Human pharmacokinetic prediction results for 14 compounds

Parameter	Group	T_1/2_						CL_tot_					
			
		6 organs		8 organs		10 organs		6 organs		8 organs		10 organs	
							
		+	-	+	-	+	-	+	-	+	-	+	-
AFE	acidic	5.45	38.7	2.26	16.6	1.36	13.5	1.39	305	1.07	26.5	2.56	14.7
	neutral	3.58	36.6	2.97	2.12	1.64	2.25	4.01	68.5	1.47	14.8	1.38	10.8
	basic	4.13	36.8	1.85	7.48	1.76	6.28	1.34	24.4	1.62	5.64	1.10	4.37
	zwitterionic	2.80	14.4	1.41	5.10	1.36	3.85	1.40	33.3	1.48	7.40	1.63	5.67

AFE	Overall	3.35	23.2	1.75	5.78	1.48	4.74	1.63	47.4	1.47	9.24	1.56	6.74
within-2-fold (%)		0	0	62	14	93	14	92	0	92	0	79	0

+: WBPBPK model with SAC, -: WBPBPK model without SAC.

**Table 3:** AFE values and within-2-fold errors from the present study and previous studies

	Method	n	AFE		Within-2-fold error (%)	
				
			T_1/2_	CL_tot_	T_1/2_	CL_tot_
Present work	SAC-WBPBPK	14	1.5	1.6	93	79

	generic WBPBPK	19^7)^	2.2	2.7	71	71
		19 or 26^8)^	1.5	1.1	69	74
Previous work	*in silico*^61)^	18	N/A	2.8	N/A	50
	animal scale-up	19^1)^	2.4	3.4	53	37
		18^62)^	N/A	2.5	N/A	50

N/A: not available.

**Figure 4:**
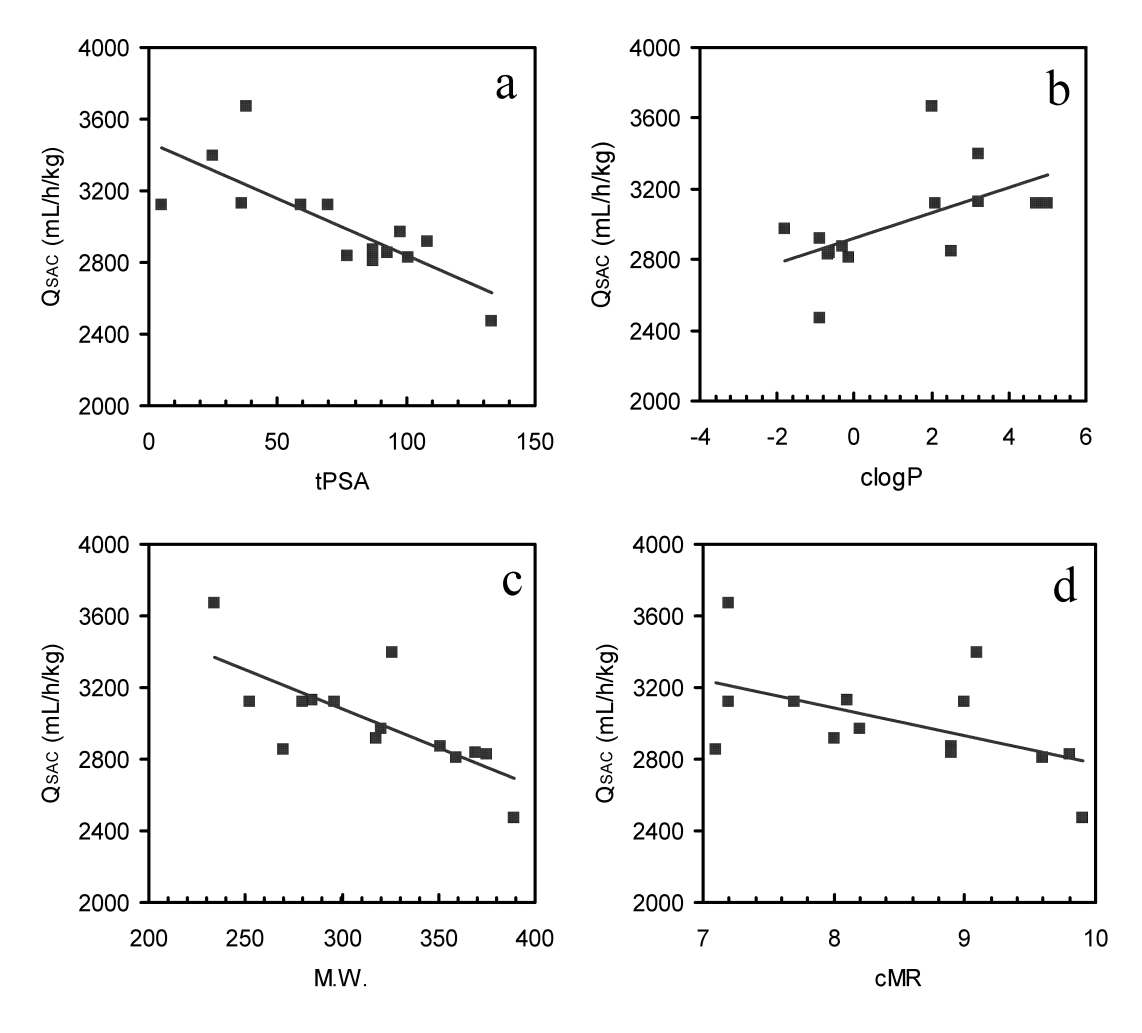
**Correlation of Q_SAC _with physicochemical parameters**. (a) tPSA, (b) clogP, (c) M.W., (d) cMR. The solid lines represent regression.

Significant correlations or non-significant trends were observed between Q_SAC_, the blood flow rate of a SAC (Table 4), and four physicochemical parameters (tPSA, clogP, M.W. and cMR). The correlation coefficients between Q_SAC _and tPSA, clogP, M.W. and cMR were 0.78, 0.57, 0.73 and 0.52, respectively (Figure 4a–d).

**Table 4:** Values of Q_SAC_, V_SAC_, Kp_SAC_, and various physicochemical parameters

Compound	SAC input parameter				Physicochemical parameter				acidic/neutral/basic/zwitterionic
			
	Q	V	Kp	VKp^*a*^	M.W.	clogP	cMR	tPSA	
Tolbutamide	2850	330	0.5	165	270	2.5	7.1	93	acidic
Diclofenac	3120	300	0.3	90	296	4.7	7.7	59	acidic
Midazolam	3400	500	1	500	326	3.2	9.1	25	neutral
Diazepam	3130	380	2	760	285	3.2	8.1	36	neutral
Phenytoin	3120	5	0.3	1.5	252	2.1	7.2	70	basic
Imipramine	3120	790	8	6320	280	5.0	9.0	5	basic
Lidocaine	3670	500	2	1000	234	2.0	7.2	38	basic
Gatifloxacin	2830	800	1	800	375	-0.69	9.8	101	zwitterionic
Grepafloxacin	2810	300	1	300	359	-0.13	9.6	87	zwitterionic
Gemifloxacin	2470	100	1	100	389	-0.89	9.9	133	zwitterionic
Pazufloxacin	2920	430	1	430	318	-0.87	8.0	108	zwitterionic
Enoxacin	2970	455	5	2275	320	-1.8	8.2	98	zwitterionic
Fleroxacin	2840	100	1	100	369	-0.65	8.9	77	zwitterionic
Lomefloxacin	2870	650	6	3900	351	-0.30	8.9	87	zwitterionic

^*a*^VKp represents the product of V and Kp.

## Discussion

### Investigation of the lack of precision in simulations of human pharmacokinetics using the generic WBPBPK model

This study was conducted to clarify the main cause of the poor predictions obtained with the generic WBPBPK model and to enable a model to be constructed that could address this problem easily. We initially attempted to elucidate the divergence in the precision of predictions with the number of organs investigated, i.e. in the 6-, 8- and 10-organ models. Poor precision and discrepancies may be related to one or more of the following: active versus passive transportation systems, species differences in metabolism, and physiological factors such as blood flow rate, tissue volume and the number of organs involved. Other factors could also be involved. The results of this series are shown in Figure 2: increasing the number of organs in the model improved the precision of prediction. These results indicate that failure to account for particular physiological factors may contribute to the poor predicted values from the generic WBPBPK model.

On the basis of the present findings, we inferred that not only species differences in active transportation systems, metabolism, etc., but also failure to account for the physiological parameters of each individual and each species, were responsible for the poor predicted values by previous WBPBPK models. Therefore, the precision with which human pharmacokinetics were predicted was examined by adding a single adjusting compartment (SAC), a newly developed virtual organ that could be expected to improve the precision of predictions if added to the generic WBPBPK model. The results are shown in Figure 3. Fitting of the simulated to the observed rat pharmacokinetics before scaling up to the human was successful and the AFE values of T_1/2 _and CL_tot _were lower than 1.1 for almost all compounds. These findings supported our initial assumptions, because the improvement in precision observed with the model incorporating the SAC implicated the previous failure to account for blood flow rate, tissue volume and tissue distribution.

The parameters for elucidating the precision of prediction were calculated (Table 2): the AFEs of CL_tot _and T_1/2 _were greatly improved by incorporating a SAC into the 10-organ model. If the only major cause of poor predictive precision had been differences in the active transportation systems of different species, then it would not have been possible to correct for differences in predictive precision. However, inclusion of a SAC in the model corrected for the divergence resulting from active transportation systems and metabolism, provided that no species differences were involved. These findings did not contradict the assumptions made for the present series, because use of actual hepatic clearance values did not improve the precision of predictions. It is therefore reasonable to conclude that the poor predictive value of the previous methods is due to their failure to account for physiological factors.

The predictions of CL_tot _were less precise for tolbutamide, diclofenac, diazepam, grepafloxacin and lomefloxacin than for the other compounds tested, even when a SAC was incorporated into the 10-organ model. The divergence of prediction for the two acidic drugs is thought to have been caused by drug binding to plasma proteins, i.e. acidic drugs have a high affinity for plasma albumin, which leads to a lower contribution to tissue distribution. Consequently, most of the total pharmacokinetics of a drug can be described by a SAC and a clearance equation, together with a scaling-up equation to adjust for the results obtained from rats. However, a SAC acts only in a supporting role. The scaling-up equation also acts only in a supporting role. Therefore, the precision of prediction for the two acidic drugs tested here might have been worse than that for the other drugs. Specifically, in order to obtain precise predictions, the tissue distribution must have a large influence on the model.

Diazepam, a drug for which predictions show considerable divergence in precision, is known to be a substrate of human MDR1 [58]. Moreover, grepafloxacin is known to be a substrate of human MRP1 and rat Mrp2 [59,60]. However, there are no data regarding the contribution of rat Mdr1 to diazepam pharmacokinetics or of rat Mrp1 and human MRP2 in the case of grepafloxacin. In addition, the differences between observed and predicted values were smaller than those obtained when no SAC was incorporated. Previously reported findings, taken together with the results of the present study, indicate the involvement of both an active transportation system and species differences. However, these factors play only a minor role in the predictive precision of the generic WBPBPK model.

Table 3 compares the predictive precision of the SAC-WBPBPK model with previous methods. The best within-2-fold error for predicting human T_1/2 _values was achieved with the 10-organ model with a SAC, and the results were even better for CL_tot_. Regardless of the AFE values associated with each of the previous methods (2 in both cases), the values for T_1/2 _and CL_tot _in the SAC-WBPBPK model showed more precise predictions; both were approximately 1.5.

In summary, this series revealed that a major factor leading to the poor precision observed with the generic WBPBPK model was the failure to account for human physiological parameters. The precision of a generic WBPBPK model was improved by incorporating a SAC, which included such physiological parameters. The results also indicated that the SAC-WBPBPK model will be more useful than previous WBPBPK models for predicting human pharmacokinetics, particularly in cases when predictions are made with data obtained before the onset of clinical trials.

### Indirect investigation of the lack of precision of simulations of human pharmacokinetics using SAC-related parameters

The input parameters for the SAC in this study were useful not only in terms of fitting the data to rat pharmacokinetics, but also for investigating factors that were missing from previous models. Initially, it was confirmed that Q_SAC_, V_SAC _and Kp_SAC _each correlated with various physicochemical parameters (Table 4). Significant correlations were confirmed between Q_SAC _and three physicochemical parameters (topological polar surface area (tPSA), molecular weight (M.W.), and calculated logP (clogP)) and a non-significant trend was observed between Q_SAC _and calculated molecular reflectability (cMR) (Figure 4). In particular, for the correlations between Q_SAC _and tPSA, a negative slope below the 0.1% significance criterion was observed. Generally, compounds with larger tPSA values are known to permeate the cell membrane with more difficulty. The finding of large Q_SAC _values indicated that the previous WBPBPK model does not take sufficient account of organs with high blood flow rates. On the other hand, small Q_SAC _values indicate that the previous model was unable to account for organs with low blood flow rates. The incorporation of a SAC in the model improved this issue. The negative-slope correlation between Q_SAC _and tPSA indicated the following: a compound with a low tPSA value (i.e. a compound that easily permeates the cell membrane and is therefore readily distributed among tissues) does not account for the factor of relative blood flow rate. Thus, high blood flow rates could affect the pharmacokinetics of such a compound because cell membrane permeation is not a major factor. Accordingly, it is reasonable to assume that the physiological factor of blood flow rate, such as blood flow-rate limitation, is related to the outcomes obtained from models. In contrast, for compounds associated with large tPSA values, membrane permeability contributes more than blood flow rate because permeability is low. The problem caused by a large Q_SAC _(small tPSA) could be resolved by incorporating a membrane permeation process into the WBPBPK model. However, the problem caused by a small Q_SAC _(large tPSA) cannot be resolved easily: it is difficult to choose an adequate blood flow rate for each model because of variation among individuals. This factor could be the cause of poor predictions for large Q_SAC _drugs. Therefore, we should keep these points in mind when we perform a proper human pharmacokinetics simulation. In short, previous models did not sufficiently account for the relationship between physiological factors and the unique distribution that is caused by an individual compound's physicochemical properties. Moreover, adding considerations such as a permeation process and individual differences in blood flow rate for constructing a generic WBPBPK model could improve the precision of prediction.

The significant correlations that we found between clogP and Q_SAC _are also considered reasonable, as was the case with tPSA, because when a drug is more lipophilic, its ability to permeate the cell membrane increases, resulting in a smooth distribution to certain tissues. Moreover, this factor is not related to the presence of an active transportation system. However, the simple incorporation of organs did not account for a precise system, because drug metabolism contributes more when lipophilicity increases. On the other hand, the present findings indicate that differences in active transportation systems and metabolism between species did not play a major role in the model's predictions; the improvement in predictive precision when correcting for physiological factors by incorporating a SAC played a larger role. These conclusions were supported by the correlation between Q_SAC _and M.W., and by the tendency of Q_SAC _and cMR to reflect molecular size. Q_SAC _and cMR showed no significant correlations. However, the bias of cMR values of selected compounds in this study could explain why no significant correlations were found. The correlation between Q_SAC _and cMR could be significant, provided the number of test compounds was increased. These results indicate that physiological limitations such as blood flow and membrane permeability were involved in improving the predictive precision of the WBPBPK model. Furthermore, such physiological limitations were not accounted for sufficiently in previous WBPBPK models.

No significant correlations were observed between V_SAC _or Kp_SAC _and the physicochemical parameters. However, V_SAC _and Kp_SAC _tended to overestimate T_1/2 _as the values increased (data not shown). Moreover, the tendency toward overestimation was especially marked when the product of V_SAC _and Kp_SAC_, which represented the degree of tissue distribution, was considered. These results indicate that the SAC was incorporated into this WBPBPK model as an organ with relatively slow drug transportation and slow drug elimination. Therefore, estimates of T_1/2 _tended to be longer when more of the drug is distributed to a SAC. With regard to the generic WBPBPK model without a SAC, the precision of prediction of T_1/2 _was relatively good. However, the prediction of CL_tot _showed low precision. From these results, it is possible that the volume of distribution (Vd) value was not accurately predicted. This assumption indicates that the related factors V_SAC _and Kp_SAC _in the SAC-WBPBPK model were not present in the previous generic WBPBPK model because, fundamentally, Vd is predicted using organ volumes and the Kp value of each organ. In the present study, the Kp value was not corrected by the blood free fraction (f_B_) in rat or human when the model was constructed. Therefore, the actual Kp values for humans were different from the experimental values for the rat, which were used in the present study. Moreover, inter-individual differences in organ volume are not considered in the generic WBPBPK model. Accordingly, organ volume as a physiological parameter should have been accounted for in more detail, including the inter-individual variability of the data set, as well as drug-specific parameters such as Kp values.

The addition of a SAC, such as that developed for this study, to various generic WBPBPK models may enhance the precision of human pharmacokinetics simulations. This approach may also facilitate with the handling of certain species differences (e.g. intrinsic clearance) because the SAC can be used as the "rest of the body (carcass)", i.e. as a non-specific compartment. Furthermore, this approach did not require arbitrary alterations of the actual experimental data, which distinguishes it from methods in which the observed data must be altered to fit the animal (rat) findings. Thus, the present approach is a more rational methodology for prediction. In this regard, we will discuss the concept underlying the model presented here. Dedrick's animal scaling-up is an empirical approach. In contrast, a WBPBPK model entails a mechanistic approach. However, the generic WBPBPK model, which has been used at the preclinical stage, contains empirical factors such as Kp values, and a clearance prediction method for scaling up to the human. Moreover, if membrane permeation processes are incorporated into the model, we have to rely on empirical methods to scale up to human permeation rate constants. Nevertheless, the generic WBPBPK model is applicable for predicting human pharmacokinetics. That is because almost all parts of this system consist of actual human physiological parameters and are linked mechanistically. Therefore, the WBPBPK approach can elucidate kinetics in organs and is applicable for a variety of uses. The SAC approach is a hybrid of an empirical and a mechanistic approach. Using a SAC, we found that the primary cause of poor prediction was a failure to consider physiological systems. Therefore, a SAC approach is compatible with a mechanistic approach because it complements previous problems. On the other hand, a SAC is not just described as a physiological system. In this context, it is more empirical than the generic WBPBPK model used previously. However, despite including an empirical factor, the SAC-WBPBPK model is more rational than the previous generic WBPBPK models. Moreover, our model addresses the cause of poor prediction in previous generic models, and does not need to manipulate observed experimental values to adjust to rat pharmacokinetics.

Some limitations are associated with the addition of a SAC. In this study, tolbutamide kinetics could only be simulated in a 10-organ model. If no upper or lower limits could be set as input parameters for a SAC, then the model would be unable to deal adequately with outliers. This problem has not yet been resolved, even when corrections were made using a scaling-up equation for human Q_SAC_. This matter will require further study.

## Conclusion

Incorporation of a SAC into a generic WBPBPK model, as performed in this study, significantly improved the precision of predictions of human pharmacokinetics. For the first time, failure to account for certain physiological parameters was identified as a major problem in previous generic WBPBPK models, in addition to confounders such as species differences in terms of metabolism and the presence/absence of active transportation systems (i.e. transporters). Moreover, the SAC-WBPBPK model performed better than all previous methods in terms of precision of prediction. Moreover, this newly developed model entails a simpler and more straightforward methodology than older models. It is likely that the present model will be useful not only for predicting clinical pharmacokinetics, but also for analyzing PBPK/PD at the preclinical stage in simulations of drug efficacy.

## Competing interests

The authors declare that they have no competing interests.

## Authors' contributions

HA conceived the idea, developed the model, performed the analysis, and drafted the manuscript. SI and WH assisted in the analysis and preparation of the manuscript. IN assisted in the analysis and preparation of the manuscript, and participated in helpful discussion. All authors read and approved the final manuscript.
