# Correction: Utility of a single adjusting compartment: a novel methodology for whole body physiologically-based pharmacokinetic modelling

**DOI:** 10.1186/1742-4682-6-29

**Published:** 2009-12-08

**Authors:** Hirotaka Ando, Shigeru Izawa, Wataru Hori, Ippei Nakagawa

**Affiliations:** 1 Discovery Research Laboratories, Kyorin Pharmaceutical Co, Ltd, Tochigi, Japan

## Abstract

After our work was published, we found that some of the terms in the equations were incorrect and that there were some typographical errors in the abbreviations.

In the section 'Single adjusting compartment' in Materials and Methods, VS should be VSAC.

In the last paragraph of Results, QSAC should be Q_SAC_.

The correct equations are included in this article.

These corrections will not affect the results of this study.

## Correction

After our work was published [1], we found that some of the terms in the equations were incorrect and that there were some typographical errors in the abbreviations.

In the section 'Single adjusting compartment' in Materials and Methods, V_S _should be V_SAC_.

In the last paragraph of Results, QSAC should be Q_SAC_.

The correct equations are as follows:(2)

These corrections will not affect the results of this study.

## Competing interests

The authors declare that they have no competing interests.

## References

[B1] Ando H Izawa S Hori W Nakagawa I Utility of a single adjusting compartment: a novel methodology for whole body physiologically-based pharmacokinetic modeling Theoretical Biology and Medical Modelling 2008 5 19 10.1186/1742-4682-5-19 18687151 PMC2542356

